# Predicting Cognitive Functioning for Patients with a High-Grade Glioma: Evaluating Different Representations of Tumor Location in a Common Space

**DOI:** 10.1007/s12021-024-09671-9

**Published:** 2024-06-20

**Authors:** S. M. Boelders, W. De Baene, E. Postma, K. Gehring, L. L. Ong

**Affiliations:** 1 Department of Neurosurgery, Elisabeth-TweeSteden Hospital, Tilburg, The Netherlands; 2 Department of Cognitive Sciences and AI, Tilburg University, Tilburg, The Netherlands; 3 Department of Cognitive Neuropsychology, Tilburg University Tilburg, Warandelaan 2, P. O. Box 90153, Tilburg, 5000 LE The Netherlands

**Keywords:** Cognitive functioning, High-grade glioma, Individual predictions, Lesion location, Parcellations, Dimensionality reduction

## Abstract

Cognitive functioning is increasingly considered when making treatment decisions for patients with a brain tumor in view of a personalized onco-functional balance. Ideally, one can predict cognitive functioning of individual patients to make treatment decisions considering this balance. To make accurate predictions, an informative representation of tumor location is pivotal, yet comparisons of representations are lacking. Therefore, this study compares brain atlases and principal component analysis (PCA) to represent voxel-wise tumor location. Pre-operative cognitive functioning was predicted for 246 patients with a high-grade glioma across eight cognitive tests while using different representations of voxel-wise tumor location as predictors. Voxel-wise tumor location was represented using 13 different frequently-used population average atlases, 13 randomly generated atlases, and 13 representations based on PCA. ElasticNet predictions were compared between representations and against a model solely using tumor volume. Preoperative cognitive functioning could only partly be predicted from tumor location. Performances of different representations were largely similar. Population average atlases did not result in better predictions compared to random atlases. PCA-based representation did not clearly outperform other representations, although summary metrics indicated that PCA-based representations performed somewhat better in our sample. Representations with more regions or components resulted in less accurate predictions. Population average atlases possibly cannot distinguish between functionally distinct areas when applied to patients with a glioma. This stresses the need to develop and validate methods for individual parcellations in the presence of lesions. Future studies may test if the observed small advantage of PCA-based representations generalizes to other data.

**Supplementary Information:**

The online version contains supplementary material available at 10.1007/s12021-024-09671-9.

## Introduction

Cognitive impairments are common for patients with a glioma (Acevedo-Vergara et al., 2022; Rijnen et al., 2020a; Tucha et al., 2000) and can contribute to decreased quality of life (Nassiri et al., 2019; Zamanipoor Najafabadi et al., 2021), decreased functional independence (Noll et al., 2018), and impaired medical decision-making capacity (Pace et al., 2020). Moreover, cognitive impairments are reported as a great burden for patients and caregivers (Zucchella et al., 2013).

Cognitive impairments have been related to the volume of the tumor (Karunamuni et al., 2020; Noll et al., 2016; van Kessel et al., 2019) and its location (Karunamuni et al., 2020; Rydelius et al., 2020). Both the location and size of a tumor determine its local and global effects on the brain (Dadario et al., 2021; Hart et al., 2019). Locally, a tumor may infiltrate or compress areas responsible for certain functions (Banerjee et al., 2015). Globally, a tumor may disrupt functional networks (De Baene et al., 2019a, b; Silvestri et al., 2022) by infiltrating or compressing a specific functional area, a hub region that coordinates processing in brain networks (Reber et al., 2021), or a white matter tract that connects different functional regions (Latini et al., 2021; Liu et al., 2020). Moreover, the tumor may cause high intracranial pressure impacting cognitive functioning.

Given the large number of studies relating tumor location to pre-operative cognitive functioning, one may be inclined to assume that preoperative cognitive functioning can be inferred from tumor location. This, however, may not be the case as good explanatory models do not always provide good predictions (Shmueli, 2010). Therefore, the current study sets out to test how well cognitive functioning can be predicted for unseen patients based on tumor location. Moreover, being able to predict cognitive functioning before surgery is an important first step towards predicting cognitive functioning for unseen patients after surgery. Such predictions after surgery may improve decision-making in view of a personalized onco-functional balance (Mandonnet & Duffau, 2018).

The location of a tumor is often defined in terms of the voxel-wise segmentation of the tumor. This voxel-wise segmentation is defined as a matrix (3D grid) that represents the space in the MRI scanner. This matrix consists of voxels where each voxel either is or is not part of the segmented tumor. In other words, the voxel-wise segmentation is a binary mask comprising ones for the voxels where the tumor is located and zeros everywhere else. This representation of tumor location, however, is very high-dimensional as many voxels make up this grid (e.g. 189 × 233 × 197 = 8.675.289 voxels). In machine learning, such high-dimensional representations suffer from the curse of dimensionality, complicating prediction (Mwangi et al., 2014). To counter the curse of dimensionality, researchers generally use dimensionality reduction techniques that create a mapping from a high-dimensional space to a low-dimensional space (see, e.g., van der Maaten et al., 2007). In what follows, we describe two dimensionality reduction methods that have been used in the literature to obtain a low-dimensional representation of tumor location based on voxel-wise tumor segmentation.

First, representations of tumor location based on different population average atlases have been used (e.g., the Brainnetome, AAL, or Yeo atlases). Atlases have shown to be an essential tool in neuroscience to describe characteristics of regions of interest and are regularly used as a biologically (anatomical or functional) meaningful way to reduce dimensionality (Eickhoff et al., 2018). Similar to voxel-wise segmentations, a population average atlas is defined as a matrix (3D grid) that represents the space in the MRI scanner. This matrix, however, consists of voxels where each voxel is a number between zero and the number of regions in the atlas. Here, the value of a voxel represents the region that in an average healthy sample would be located at this voxel, with zeros in all other areas (background/unlabeled). Population average atlases allow us to relate the voxels representing the segmented tumor to the corresponding brain regions in an average healthy brain. Such a representation is generally created by registering both the patient MRI including the segmentation of the tumor and the atlas to a common space such as the MNI (Montreal Neurological Institute) space. When registered to a common space, one can calculate the voxel-wise overlap of the segmentation with the different regions in the atlas. This yields a low-dimensional numerical representation, i.e., a vector with the percentage of overlap for each region. An overview of the numerous examples of studies employing population average atlases to represent tumor locations is provided by Germann et al. (2022), and such representations of tumor location have been related to cognitive functioning using lesion-symptom mapping (e.g., De Baene et al., 2019a). A variety of different population atlases exist, differing in the number of regions defined and the included anatomical structures. Moreover, different atlases are defined based on either anatomy/cytoarchitecture, white matter connectivity between regions which represents the inter-regional structural connectivity, functional connectivity between regions which represents global function, or combinations thereof (Eickhoff et al., 2018).

Second, representations of tumor location based on principal component analysis (PCA) have been used. PCA is a popular method for dimensionality reduction that results in a low-dimensional space consisting of uncorrelated features that capture as much variance in the input data as possible given the constraint of being a linear model. Representations based on PCA have been used to predict cognitive functioning in patients with a glioma (Zangrossi et al., 2022) and in patients who suffered from a stroke (Facchini et al., 2023; Ramsey et al., 2017; Salvalaggio et al., 2020; Siegel et al., 2016). PCA works well when the features in the original data are correlated. For voxel-wise tumor location, this is the case for three reasons. First, neighboring voxels are correlated as they are generally part of the same tumor segmentation (spatial continuity). Second, the spatial distribution of primary brain tumors is not random (De Leeuw et al., 2019; Numan et al., 2022; Tang, 2017) and can be related to, for instance, molecular subgroups and connectomics (Mandal et al., 2020; Numan et al., 2022; Romero-Garcia et al., 2023; Wijnenga et al., 2019). Third, the growth pattern of tumors themselves is not random (Claes et al., 2007), with tumor cells migrating faster along the direction of fiber tracts (Swanson et al., 2000). Given that PCA represents as much variance in as few uncorrelated features as possible, we hypothesize that representations using PCA allow for better predictions when compared to using population average atlases.

A recent study by Revell et al. (2022) compared different population average atlases against random atlases. Random atlases are comparable to population average atlases, except that the boundaries of regions are defined at random, and therefore do not carry any biological meaning. The creation of random atlases will be explained in the method section (subsection ‘representations of tumor location’). Their results showed that many population average atlases commonly used in the neuroscience literature result in comparable effect sizes when compared to random atlases (when testing specific hypotheses about epilepsy pathophysiology). Their results lead us to hypothesize that the choice of a population average atlas used to represent tumor location is of little influence when predicting cognitive functioning for patients with a glioma.

To our knowledge, no studies exist that systematically compare population average atlases, PCA, and random atlases as lower-dimensional representations of voxel-wise tumor location. Therefore, the current study compared these representations for the prediction of pre-operative cognitive functioning of 246 patients with a high-grade glioma. Based on our findings, we aim to make recommendations regarding the most suitable representations of tumor location when predicting post-operative cognitive functioning.

## Methods

### Design

#### Participants

Patients with grade 3 or 4 Glioblastoma, Astrocytoma, and Oligoastrocytoma who were scheduled for surgery at the Elisabeth-TweeSteden Hospital, Tilburg, The Netherlands, and underwent pre-operative cognitive screening as part of clinical care between 2010 and 2019 were included. Here, WHO-grade was defined according to the standard of care at the time of treatment. Patients were not included when they were under 18, had a progressive neurological disease, had a psychiatric or acute neurological disorder within the past two years, or had reduced testability for the neuropsychological assessment.

#### Neuropsychological Assessment and Collection of Sociodemographic Variables

Cognitive screening was done using the computerized CNS Vital Signs (CNS VS) (CNS Vital Signs, n.d.) test battery, comprising a verbal memory recognition test, visual memory recognition test, symbol digit coding test, Stroop test, shifting attention test, continuous performance test, motor speed test, and a measure of reaction time. The psychometric properties of this battery were demonstrated to be comparable to the pen-and-paper tests, on which they were based, in pediatric patients (Plourde et al., 2018), patients with various neuropsychiatric disorders, and healthy participants (Gualtieri & Johnson, 2006).

A well-trained technician, a neuropsychologist (in training), provided instructions to patients prior to each test and subsequently recorded the validity of performance for every test. Considerations for the validity of a test are the patient understanding the test, showing sufficient effort, having no vision or motor impairments that might affect the test, and the absence of any distractions. Test scores deemed invalid were excluded from the current analyses on a test-by-test basis. The CNS VS test battery and administration of two additional pen and paper tests (not included in the current analyses) took approximately 30 to 40 min to complete. For all participants, a standardized interview was performed beforehand to obtain demographic variables such as age, sex, and education. Education was recorded on the Dutch Verhage scale (Verhage, 1965).

#### Test Measures and Standardization

From the CNS VS test outcomes, eight test scores were calculated as specified in Appendix 1. Subsequently, test scores were converted into socio-demographically adjusted z-scores by correcting for effects of age, sex, and education as found in the data of normative controls using a multiple regression approach (Rijnen, 2017). Normalization of test scores was done relative to healthy participants, where z-scores of healthy participants were set to have zero mean and unit variance.

#### Image Processing and Segmentation

All patients had a presurgical T1, gadolinium-enhanced T1, T2, and FLAIR MRI, or a subset thereof. Scans were collected using a 3T Philips Achieva, 1.5T Philips Intera, or 1.5T Philips Ingenia scanner. The median voxel size was (0.80 × 0.80 × 0.80mm) and all voxel sizes were in the range (0.68 × 0.68 × 0.8mm – 1.00 × 1.00 × 1.00mm).

Patient MRIs were resliced to an isotropic 1 mm resolution and registered to MNI space (comprising 189 × 233 × 197 voxels) using Regaladin (Ourselin et al., 2001) from the NiftyReg package. Linear registration was used which was found to perform equally well as non-linear registration for patients with a brain tumor (Visser et al., 2020). Afterwards, skull stripping was performed using HD-BET which is designed to be robust to a variety of different lesions (Isensee et al., 2019). Registration was performed before skull-stripping which is in line with the Cancer Imaging Phenomics Toolkit (Davatzikos et al., 2018) as used for the BraTS challenges since 2017 (Adewole et al., 2023).

The contrast-enhancing region of the tumors was segmented using a convolutional neural network with a U-Net architecture. Two different models were used for segmentation and the best segmentation was selected manually for each patient. Models used were nnU-Net (Isensee et al., 2021) as trained on T1, T1c, T2, and Flair scans from the BraTS dataset or subsets thereof (Bakas et al., 2019; Menze et al., 2015) and AGU-Net as available in the Radionics tool using T1c images (Bouget et al., 2022). Automatic segmentations were manually validated and incorrect segmentations were re-segmented semi-automatically using the snake tool in ITK-Snap (Yushkevich et al., 2006). The segmentation results were validated and corrected by the first author (SB) under the supervision of an experienced neurosurgeon.

#### Representations of Tumor Location

Each high-dimensional voxel-wise segmentation in MNI space was reduced to 39 different lower-dimensional representations. This was done using population average atlases, random atlases, and PCA. In what follows, we describe how these three types of representations were obtained.

Population average atlas-based representations were created for 13 different **population average atlases**. These representations were created by overlaying the population average atlas with the voxel-wise tumor segmentation (both in MNI space). Next, for each region in the atlas, the number of voxels of this region that overlap with the voxel-wise tumor segmentation was counted and divided by the total number of voxels making up that region. This resulted in a representation that was a vector with a length equal to the number of regions. In this vector, each value represented the percentage of this region that was covered by the tumor segmentation. The atlases used were the standard set of ten population average atlases as proposed and provided by Revell et al. (2022), except for the Destrieux atlas as it requires a cortical surface model (Fischl et al., 2004). In addition to this standard set of atlases, we added three additional atlases: the MNI structural atlas with nine regions (Mazziotta et al., 2001), this same atlas where all regions were separated by hemisphere resulting in eighteen regions, and an atlas only representing the two hemispheres (consisting of all voxels in the MNI structural atlas). These additional atlases were added to study to assess the amount of variance that can be explained using atlases that only provide a rough description of tumor location. The complete set of atlases used in the current study including their characteristics are described in Appendix 2.

As differences in the amount of variance explained between atlases may be due to the number of areas defined in an atlas (its dimensionality) instead of the biological meaning of the regions themselves, we additionally included 13 **random atlases**. The random atlases were generated using an algorithm similar to the grassfire algorithm (Blum, 1967), in line with the work of Revell et al. (2022) and Zalesky et al. (2010), and were created to cover the same voxels as the MNI structural atlas (Mazziotta et al., 2001). The algorithm randomly selects *N* seed voxels within the voxels we want our atlas to cover. Each seed voxel iteratively ‘grows’ into a region of the atlas by adding neighboring voxels until we end up with *N* regions covering the voxels we want our atlas to cover. Thirteen different random atlases were created, each with its number of regions matching those in a corresponding population average atlases to facilitate comparison among representations. Random atlases were created thirty times with the same number of regions, each of which was individually used to predict cognitive function. This resulted in 30 random atlases for each of the 13 different dimensionalities (numbers of regions*).* The performance of random atlases was defined as the median performance of the thirty random atlases with the same number of regions. By taking the median performance of these random atlases which all stemmed from randomly selected seed voxels, we minimize the chance that the obtained performance was due to obtaining a functional region by chance. The median and interquartile range were used as the performances were not normally distributed due to the definition of the R2 score which allows it to range between minus infinity (extremely poor predictions) and one (perfect predictions).

**PCA-based representations** were created by performing PCA on the voxel-wise tumor segmentations. PCA learns several components, that map the high-dimensional matrix representing the voxel-wise tumor segmentations to a low-dimensional representation comprising a number of orthogonal features equivalent to the number of patients (and thus segmentations) in the sample. In the current study, this mapping was learned using a combined set comprising segmentation of high-grade glioma as available in the BraTS2019 (n = 259) (Bakas et al., 2017, 2019; Menze et al., 2015) dataset and the UCSF dataset (n = 438) (Calabrese et al., 2022), excluding patients with a low-grade glioma. Next, these mappings were truncated to the desired number of features by retaining only the first components, each of which explains progressively less variance than the previous one. The resulting mappings were used to obtain the PCA-based representations for patients in our current sample. The numbers of features maintained were equal to the numbers of regions in the 13 different population average atlases and random atlases to facilitate comparison. PCA was fitted on an external dataset instead of our current sample to prevent the PCA solution from including information regarding the data point that we are predicting*,* i.e., to prevent data leakage. PCA was implemented using the PCA function in Scikit-Learn (v1.1.1, implemented using Singular Value Decomposition). To interpret the full and reduced PCA-based representations (which only differ in the number of features maintained, respectively), the amount of variance explained was plotted relative to the number of features maintained. Moreover, the components were visualized in MNI space by, for each component, plotting the magnitude with which each voxel loaded on the corresponding feature.

### Predicting Cognitive Function

The eight test scores representing pre-operative cognitive functioning were predicted while using the different representations of tumor location. Prediction accuracies were compared to answer seven different questions (described below).

For prediction, the ElasticNet model (Hastie et al., 2009) was used as it can handle a large number of predictors relative to the sample size. This is possible through regularization of both the number of predictors used (L1/Lasso regularization) and the magnitude of its coefficients (L2/Ridge regularization). Prediction performance was evaluated using leave-one-out cross-validation. The hyperparameters configuring the amount of regularization applied and the balance between L1 and L2 regularization were set using sixfold cross-validation. Both normalization (zero mean, unit variance) and the selection of hyperparameters were performed within the leave-one-out cross-validation loop to obtain an unbiased performance estimate (Vabalas et al., 2019). Models were fitted to reduce the mean squared error and performance was reported as the amount of variance explained. For random atlases, predictions were performed individually for the 30 different random atlases with the same number of regions. The Elastic Net implementation from Scikit-learn (V1.1.1) was used.

The previously described representations of voxel-wise tumor location all contain information regarding the **tumor volume** (i.e. the total overlap with all different regions in an atlas). Therefore, prediction models were compared against models using solely tumor volume as a predictor. This is equivalent to using an atlas with one region covering the complete brain because the number of overlapping tumor voxels is proportional to tumor volume. Throughout the rest of this study, we refer to the prediction model using only tumor volume as the baseline model.

### Comparing Different Representations

To provide an intuitive overview of all prediction results, the variance explained for the eight test scores given the different representations relative to the dimensionality of the representation was visualized, separately for each test. For random atlases and PCA-based representations, the performance was visualized as linearly interpolated curves representing the variance explained as a function of dimensionality. For random atlases, the line is defined as the median performance of the 30 different atlases with the same number of dimensions. Moreover, the interquartile range is visualized to indicate the variability in performance between different random atlases. To improve the resolution of the interpolated lines, seventeen additional dimensionalities were included for these representations, where the number of regions in these atlases was picked at fixed intervals from a log scale ranging between the number of regions in the smallest and largest atlas, i.e., 2 and 360, respectively.

Based on these same results (excluding the additional dimensionalities used for the visualization), different summary metrics of the results were created to answer the following seven questions.

1. To what extent does tumor volume allow for the prediction of cognitive test scores?
  To answer this question, the amount of variance that can be explained using the baseline model was reported individually for each test. The baseline model uses only tumor volume as a predictor, which is equivalent to using an atlas comprising one region covering the whole brain.
2. Which representation(s) result(s) in the best predictions per test score?
  The top three most predictive representations per cognitive test were reported. Performance was reported both as the variance explained and the differences in variance explained between the representation and the baseline model.
3. Do representations of location improve performance over the baseline model?
  Two summary metrics were calculated to describe the performance per type of representation (population average atlases, random atlases, and PCA-based representations) relative to the baseline model: one summarizing the results per cognitive test, and one summarizing the results per dimensionality of the representations. To summarize the results per test, for each test the number of dimensionalities was counted for which a representation of tumor location resulted in a better performance compared to the baseline model. To summarize the results per dimensionality, for each dimensionality, the number of tests was counted on which a representation given a dimensionality performed better than the baseline model.
4. Does using population average atlases result in better predictions compared to using random atlases?
  Again, two summary metrics were calculated similar to question 3. To summarize the results per test, for each cognitive test the number of dimensionalities was counted for which population average atlases outperformed random atlases. To summarize the results per dimensionality, for each dimensionality, the number of cognitive tests was counted on which a given atlas performed better than a random atlas with the same number of regions.
5. Does using PCA-based representations result in better prediction compared to random atlases?
  The same summary metrics as for population average atlases (question 4) were calculated, using PCA-based representations instead of population average atlases.
6. Does using PCA-based representations result in better predictions compared to population average atlases?
  Again, two metrics were calculated. To summarize the results per cognitive test, for each test the number of dimensionalities was counted on which PCA-based representations performed better than population average atlases. To summarize the results per dimensionality, the number of cognitive tests was counted on which PCA-based representation performed better than the population average atlas with the same number of regions.
7. Given an atlas, would a different representation with the same number of dimensions result in better predictions?
  The best-performing representation given a combination of dimensionality and cognitive test was reported.

All Python code used in the current study is made available as online supplementary material.

## Results

### Baseline Characteristics

**Fig. 1:**
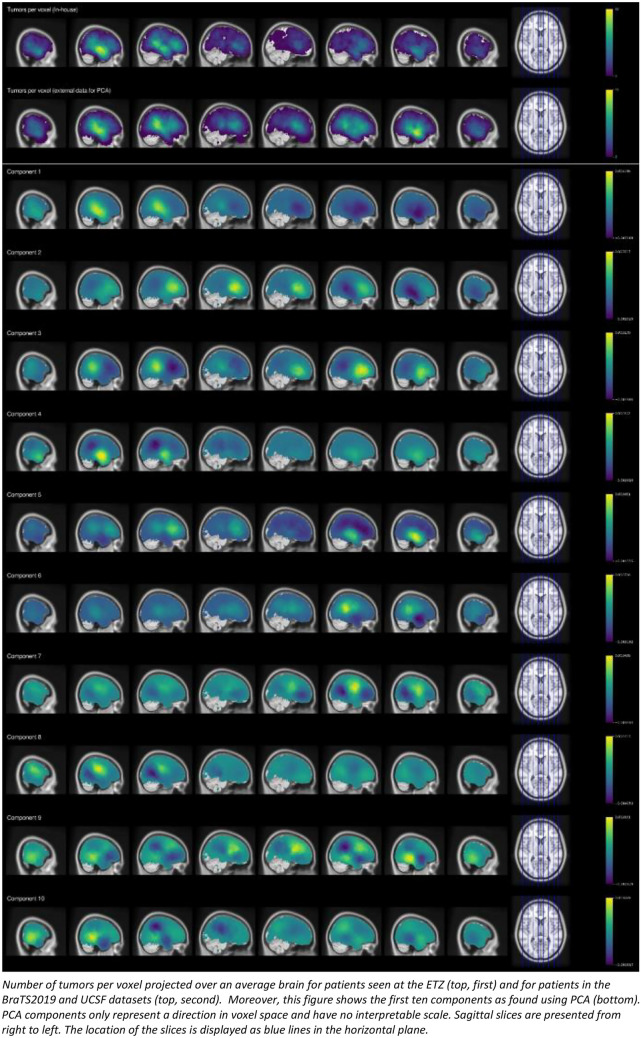
Distribution of tumors and the first ten PCA components

Sample characteristics including the location of tumors as defined using the MNI structural atlas split by hemisphere are provided in Table 1. The number of tumors per voxel in MNI space is visualized Fig. 1 (top) both for the in-house dataset (first row) and the combined sample comprising the high-grade glioma from the BraTS2019 and UCSF datasets (second row). In this figure, the eight columns describe eight sagittal slices (starting at the right hemisphere).

**Table 1:** Sample characteristics

	count	mean	std	min	25%	50%	75%	max
Age	246	57.2	12.45	18.00	51.00	59.00	67.00	81.00
Sex (m)	246	69.1%						
Education Verhage	246	4.9	1.17	1.00	4.00	5.00	6.00	7.00
Glioblastoma	246	86.6%						
Astrocytoma	246	9.4%						
Oligodendroglioma	246	3.3%						
Oligoastrocytoma	246	0.8%						
WHO grade 3 (vs 4)*	246	13.8%						
Left lateralized	246	37.8%						
Right lateralized	246	63.0%						
Bilateral	246	0.8%						
Tumor volume (cm^3^)	246	51.04	37.17	0.31	23.30	42.54	71.23	206.97
Verbal memory (Z-score)	232	-0.59	1.29	-3.24	-1.44	-0.45	0.37	2.04
Visual memory (Z-score)	246	-0.55	1.63	-3.94	-1.73	-0.40	0.70	2.36
Symbol digit coding test (Z-score)	246	-1.00	1.51	-3.82	-2.10	-1.00	0.09	2.36
Stroop interference ratio (Z-score)	223	0.18	1.34	-3.00	-0.61	0.09	1.05	3.62
Simple reaction time (Z-score)	241	-1.64	2.41	-7.68	-2.50	-0.89	0.15	1.47
Continuous performance test (Z-score)	246	-0.78	1.54	-4.25	-1.66	-0.66	0.33	2.15
Shifting attention test (Z-score)	222	-0.75	1.05	-2.35	-1.60	-0.87	-0.01	2.96
Finger tapping test (Z-score)	222	-0.94	1.40	-4.28	-1.86	-0.81	0.02	2.68
Left Caudate	246	4.0%	14.0%	0.0%	0.0%	0.0%	0.0%	89.1%
Left Cerebellum	246	0.0%	0.1%	0.0%	0.0%	0.0%	0.0%	1.2%
Left Frontal Lobe	246	1.6%	4.3%	0.0%	0.0%	0.0%	0.6%	29.1%
Left Insula	246	5.2%	14.3%	0.0%	0.0%	0.0%	0.0%	98.6%
Left Occipital Lobe	246	0.7%	3.2%	0.0%	0.0%	0.0%	0.0%	26.6%
Left Parietal Lobe	246	1.1%	3.6%	0.0%	0.0%	0.0%	0.0%	21.8%
Left Putamen	246	4.7%	14.4%	0.0%	0.0%	0.0%	0.0%	100.0%
Left Temporal Lobe	246	2.4%	6.9%	0.0%	0.0%	0.0%	0.0%	41.8%
Left Thalamus	246	1.2%	7.1%	0.0%	0.0%	0.0%	0.0%	96.6%
Right Caudate	246	5.3%	13.3%	0.0%	0.0%	0.0%	2.3%	73.5%
Right Cerebellum	246	0.0%	0.1%	0.0%	0.0%	0.0%	0.0%	1.1%
Right Frontal Lobe	246	2.5%	5.0%	0.0%	0.0%	0.0%	2.5%	27.5%
Right Insula	246	9.9%	18.5%	0.0%	0.0%	0.0%	12.4%	86.8%
Right Occipital Lobe	246	1.2%	4.9%	0.0%	0.0%	0.0%	0.0%	39.7%
Right Parietal Lobe	246	2.9%	6.7%	0.0%	0.0%	0.0%	1.2%	34.4%
Right Putamen	246	8.5%	19.7%	0.0%	0.0%	0.0%	4.3%	91.1%
Right Temporal Lobe	246	4.5%	9.4%	0.0%	0.0%	0.0%	2.3%	51.0%
Right Thalamus	246	3.9%	10.2%	0.0%	0.0%	0.0%	0.2%	64.3%

Sample characteristics including tumor location defined as the overlap of tumor segmentations with regions from the MNI maxprob atlas split by hemisphere. *Defined according to the standard of care at the time of treatment

To illustrate how the PCA-based representations as found using the external datasets describe tumor location, the first 10 principal components (3D matrices describing the mapping between the high- and low-dimensional representations) are visualized in Fig. 1 (bottom). The complete set of components is available as an online supplement to this study. These images show the degree to which each component relies on a given voxel in the matrix (3D grid) of voxels being part of the tumor segmentation. Here, yellow indicates a positive relation between the voxel and the component, while dark purple represents a negative relation. The first three components of the PCA solution described 20% of the variance in terms of which voxels are part of the tumor segmentation in the external dataset, and the first 10 components described over 54% of the variance. Over 70% of variance was captured by the first 57 components (for all cumulative eigenvalues, see Appendix 3).

Component 1 represents the largest source of variation in the data. The visualization of this component in MNI space reveals that it represents the distinction between the right and left temporal lobes. This is the case as the voxels in the right temporal lobe have a high value for this component (yellow) leading to a high value for the resulting feature for tumors located in this region. Similarly, the left temporal lobe has a low value (dark purple) leading to a low value on this feature for tumors located in this region. Component 2 reveals the second-largest source of variation in the data. The visualization of this component shows that it mainly distinguishes between the left-temporal and the frontal lobes as the voxels in the left-temporal lobe have a low value while the voxels in the frontal lobe have a high value. The visualization of component 3 shows that it distinguishes the right-frontal/left-parietal and left-frontal/right-parietal lobes as can be seen from the low value from the left-frontal and right-parietal lobes and the high value for the right-frontal and left-partial lobes. More generally, the first three components can be interpreted as describing the distinction between frontal, parietal, and temporal tumor locations, and allow for some separation between left and right lateralized tumors. Starting at component four, components describe more nuanced differences in location.

### Predicting Cognitive Function

**Fig. 2:**
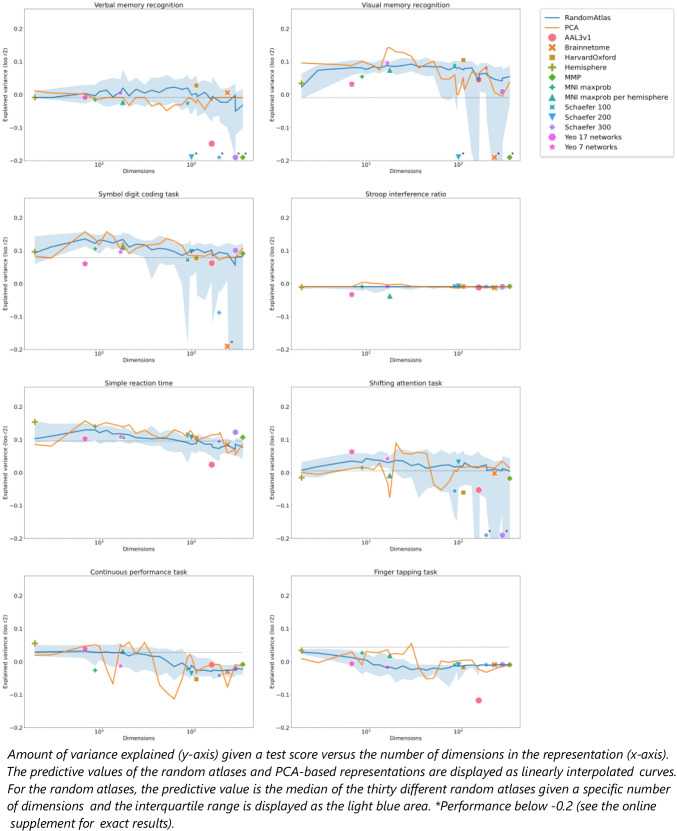
Overview of all prediction results

An overview of all prediction results is presented in Fig. 2. In this figure, the variance explained given different representations is shown against the dimensionality of the representation for each cognitive test. Population average atlases are displayed as colored markers, and random atlases and PCA-based representations are shown as blue and yellow curves respectively. These curves are linearly interpolated across the different numbers of dimensions. The blue line represents the median model performance across the 30 different random atlases given a specific number of dimensions. In addition to the median, the interquartile range is displayed as the light blue area. Finally, the dashed line represents the variance explained by the baseline model (using only tumor volume). The same results including the selected hyperparameters per model are also presented as a table as an online supplement.

From Fig. 2, six observations can be made regarding the performance across cognitive tests. First, the height of the dashed line representing the baseline model differs per cognitive test, showing that the variance explained when using only tumor volume differs per test. Second, the representation that resulted in the most variance explained (highest value on the y-axis) differed per test, showing that no representation performs best across different tests. Third, for most representations, the amount of variance explained is above the dotted line (representing the baseline model) for most cognitive tests. This indicates that using representations of tumor location generally results in a better performance when compared to solely using tumor volume (except for the continuous performance task, finger tapping task, and Stroop task, see below). Fourth, the markers representing population average atlases are scattered around the blue line representing the median performance of random atlases. This indicates that the meaning of the regions in population average atlases did not lead to a better performance when compared to random atlases (for which regions do not have any meaning). Fifth, the yellow line representing the PCA-based representations often is above all other representations. This shows that PCA-based representations generally yield the best performance. Last, we observe a negative trend regarding the dimensionality (the x-axis) of the representations with respect to performance (the y-axis). Moreover, several population average atlases and random atlases with very high dimensionality (100 dimensions and up) result in poor performance. This can be seen from the median performances of several population average atlases, and the 25th percentile (the lower side of the interquartile range) in terms of the performances of the random atlases being low (R^2^ lower than -0.1).

For individual tests, several things can be noted. First, for the finger tapping test and the continuous performance tasks the baseline model resulted in better predictions than many of the representations. This indicates that these tasks were difficult to predict from tumor location. Second, the performance when predicting the Stroop interference ratio was near zero regardless of the predictors used. This indicates that models were unable to find any relationship between the Stroop interference ratio and tumor size or location. Third, models were prone to overfit when using population average atlases or random atlases with a high number of regions when predicting verbal memory recognition, visual memory recognition, the symbol digit coding task, and the shifting attention task. For all other tasks, the models did not show signs of overfitting.

Throughout the rest of the results section, the prediction results visualized in Fig. 2 are described quantitively by calculating several summary statistics. In the next sections, the performance of a random atlas given the number of regions in the random atlas is defined as the median performance over the thirty random atlases unless stated otherwise.

1. To what extent does tumor volume allow for the prediction of cognitive functioning?
  The explained variance of the baseline model using only tumor volume is reported in Table 2 and was lower than 1% for four out of eight tests (verbal memory recognition test, visual memory recognition test, Stroop interference ratio, and shifting attention test) while explaining some variance (between 2.8% and 9.6%) on the other four tests (symbol digit coding test, simple reaction time, continuous performance test, and finger tapping test). This shows that the extent to which tumor volume allowed for predicting cognitive function differed per test with tumor volume explaining some variance on some, but not all tests.

**Table 2:** Best-performing representation of tumor location per cognitive test including the performance relative to the baseline model

**Cognitive test**	**Representation of location**	**Number of dimensions/regions**	**R**^**2**^ **(%)**	**R**^**2**^ **Baseline (%)**	**R**^**2**^ (%) improvement over baseline*
Visual memory recognition test	PCA 17	17	14.22%	-0.91%	15.13%
	PCA 18	18	14.22%		15.12%
	HarvardOxford	113	10.42%		11.33%
Verbal memory recognition test	HarvardOxford	113	2.75%	-0.87%	3.61%
	Random atlas 91	91	1.98%		2.85%
	Random atlas 18	18	1.97%		2.84%
Symbol digit coding test	PCA 7	7	15.81%	7.89%	7.91%
	Random atlas 7	7	13.58%		5.69%
	PCA 9	9	13.46%		5.56%
Stroop interference ratio	PCA 9	9	0.38%	-0.90%	1.28%
	PCA 18	18	-0.32%		0.58%
	PCA 17	17	-0.35%		0.55%
Simple reaction time	PCA 7	7	15.72%	9.56%	6.16%
	Hemisphere	2	15.39%		5.82%
	PCA 17	17	14.12%		4.56%
Shifting attention test	Yeo 7 networks	7	6.32%	0.56%	5.76%
	Yeo 17 networks	17	4.24%		3.68%
	Random atlas 7	7	3.56%		3.00%
Finger tapping test	PCA 17	17	3.64%	4.45%	-0.81%
	Hemisphere	2	3.43%		-1.02%
	PCA 7	7	2.94%		-1.51%
Continuous performance test	Hemisphere	2	5.55%	2.78%	2.77%
	PCA 9	9	5.17%		2.39%
	PCA 17	17	5.13%		2.35%

*Defined as the difference in the percentage of variance explained
2. Which representation(s) result(s) in the best predictions per test score?
  Table 2 further shows the three best-performing representations of tumor location per cognitive test. The best-performing representations explained between 0.4% and 15.7% of variance on the different cognitive tests. The improvement over the baseline model was between -0.8% and 15.1% of variance. The PCA-based representations made up 13 out of the 24 top-three representations, population average atlases made up 7 of the top-three representations, and random atlases made up 4 of the top-three representations. In terms of the top-one performing representation, 3 out of the 8 of the top-performing representations were population average atlases while the other 5 were PCA-based representations. No random atlases were amongst the top-one performing representations for any of the tests. These results show that PCA-based representations most often resulted in the best-possible, or close to best-possible predictions per test, followed by population average atlases and random atlases respectively.
  Best-performing population average atlases were the Harvard–Oxford, Hemisphere, Yeo 7 networks, and Yeo 17 networks atlases, and differed per test. Moreover, there was no clear pattern in the number of regions in population average atlases that were amongst the top-three performing representations, with the numbers of regions ranging between 2 and 113. Additionally, atlases were either based on structure or were multimodal and were either volumetric or surface-based. For PCA-based representations, all representations consisted of at most 18 components. Random atlases comprised of at most 91 regions.
3. Do representations of location improve performance over the baseline model?
  Summarizing the results per test, Table 3 shows how often a representation of location resulted in a better prediction when compared to the baseline model, individually for each cognitive test. More than half of the individual random atlases performed better than the baseline model for five out of eight tests, namely visual memory recognition, verbal memory recognition, the symbol digit coding task, the measure of simple reaction time, and the shifting attention task. This was the same for PCA-based representations. For population average atlases, this was the case for the same tests except for the verbal memory recognition and the shifting attention tasks. On average across both tests and dimensionalities, PCA performed better than the baseline in 51.0% of the cases, for random atlases this was 45.3%, and for population average atlases this was 38.5%. Representations of location rarely resulted in higher performance than the baseline model when predicting the Stroop interference ratio, the finger tapping test, and the continuous performance task (at most 23.1%, 1.0%, and 23.8% respectively across the different representations).

**Table 3:** Comparisons per cognitive test

	**Question 3: Better than baseline**			**Questions 4 and 5: Better than Random atlases**		**Question 6: Better than Population- average atlas**
Cognitive test / Representation	Random atlas (out of 390)	Population- average Atlas (out of 13)	PCA (out of 13)	Population- average Atlas (out of 13)	PCA (out of 13)	PCA (out of 13)
Visual memory recognition	**88.72%**	**76.92%**	**100.00%**	46.15%	**53.85%**	**76.92%**
Verbal memory recognition	**52.56%**	23.08%	15.38%	15.38%	46.15%	**76.92%**
Symbol digit coding task	**75.38%**	**53.85%**	**92.31%**	23.08%	23.08%	**76.92%**
Stroop interference ratio	1.28%	23.08%	23.08%	23.08%	23.08%	46.15%
Simple reaction time	**52.05%**	**76.92%**	**69.23%**	**69.23%**	**76.92%**	**53.85%**
Shifting attention task	**68.21%**	30.77%	**76.92%**	23.08%	30.77%	**69.23%**
Finger tapping task	1.03%	0.00%	0.00%	**69.23%**	46.15%	**53.85%**
Continuous performance task	23.33%	23.08%	30.77%	38.46%	**76.92%**	**76.92%**
Average	45.32%	38.46%	**50.96%**	38.46%	47.12%	**66.35%**

Results summarized over the different dimensionalities. The percentage of dimensionalities for which a representation of tumor location performed better when compared to some other representation of location. Scores indicating that over half of the dimensionalities performed better are displayed in bold
  These results show that the value of using a representation of location differed per outcome measure and that representations of tumor location regularly did not improve performance beyond those using tumor size. Moreover, these results show that PCA-based representations most consistently resulted in better predictions when compared to the baseline model. This was followed by random atlases and population average atlases respectively.
  Summarizing results per dimensionality, Table 4 shows the number of cognitive tests for which a representation with a specific number of dimensions performed better than the baseline model. Results show that random atlases performed better than the baseline model on over half of the cognitive tests for 5 out of 13 (38.5%) dimensionalities. For population average atlases this was 1 out of 13 (7.7%), and for PCA-based representations, this was 4 out of 13 (30.8%). Moreover, on average, random atlases, population average atlases, and PCA-based representations performed better than the baseline on 4.15, 3.08, 4.08 out of 8 tests respectively.

**Table 4:** Comparisons per number of dimensions

		Question 3			Question 4	Question 5	Question 6
**Number of dimensions**	Atlas	Random atlas better than baseline	Population average atlas better than baseline	PCA better than baseline	Population average atlas better than random atlas	PCA better than random atlas	PCA better than population average atlas
**2**	Hemisphere	**5**	4	3	5	2	4
**7**	Yeo 7 networks	**6**	4	**6**	2	**5**	**7**
**9**	MNI maxprob	**6**	4	**6**	2	**5**	**7**
**17**	Yeo 17 networks	**6**	**5**	**5**	3	**5**	**6**
**18**	MNI maxprob per hemisphere	**5**	4	**5**	2	**5**	**7**
**96**	OASIS DKT	4	3	4	4	3	**5**
**100**	Schaefer 100	4	3	4	3	3	**5**
**113**	HarvardOxford	4	4	4	4	2	4
**166**	AAL3v1	3	1	4	2	**5**	**7**
**200**	Schaefer 200	3	1	3	3	4	**6**
**246**	Brainnetome	3	1	3	3	2	4
**300**	Schaefer 300	3	3	3	3	3	3
**360**	MMP	2	3	3	4	**5**	4
Average		4.15	3.08	4.08	3.08	3.77	5.31

Results summarized over the eight different tests. The number of cognitive tests on which a representation of tumor location resulted in a larger amount of variance explained when compared to some other representation of tumor location, individually for each dimensionality. Representations that outperformed some other representations on more than half of the tests are displayed in bold
  Theentations of location did not always improve predictions across tests. Moreover, these results indicate that Random atlases most often result in better predictions than the baseline across cognitive tasks, followed by PCA-based representations and population average atlases respectively. An extended version of Table 4 consisting of all dimensionalities as used for Fig. 1 can be found in Appendix 4, leading to the same conclusion.
  The population average atlases that performed better than the baseline model on over half of the tests was the Yeo atlas depicting 17 different networks. For PCA-based representations, this was the case for most representations with between 7 and 18 features (or between 7 and 37 according to Appendix 4). Finally, for random atlases, this was the case for atlases with 2 up to 18 different regions (or between 2 and 65 regions according to Appendix 4).
4. Does using population average atlases result in better predictions compared to using random atlases?
  Summarizing results per test, Table 3 shows that population average atlases perform better across dimensionalities than random atlases for two out of eight tests: the measure of simple reaction time, and the finger tapping test. Table 3 further shows that on average across both tests and dimensionalities, population average atlases performed better than random atlases in 38.5% of cases. Summarizing results per dimensionality, Table 4 shows that 1 of the 13 population average atlases (7.8%) performed better than random atlases on more than half of the cognitive tests. This atlas was the hemisphere atlas comprising two regions. On average, population average atlases performed better than random atlases on 3.08 out of the 8 tests. These results show that population average atlases did not result in better predictions when compared to random atlases.
5. Does using PCA-based representations result in better predictions compared to random atlases?
  Summarizing results per test, Table 3 shows that PCA-based representations perform better on average across dimensionalities than random atlases on 3 out of 8 tests: visual memory recognition, simple reaction time, and the continuous performance task. Table 3 further shows that, across both tests and dimensionalities, on average PCA-based representations performed better than random atlases in 47.1% of cases. Summarizing results per dimensionality, Table 4 shows that for 6 out of the 13 (46.2%) dimensionalities, the PCA-based representations performed better than random atlases on more than half of the cognitive tests. Moreover, on average, PCA-based representations performed better on 3.77 out of 8 tests. These results show that PCA-based representations did not result in better predictions when compared to random atlases.
6. Does using PCA-based representations result in better predictions compared to population average atlases?
  Summarizing results per test, Table 3 shows that PCA-based representations perform better than population average atlases across dimensionalities for 7 out of 8 tests. These were all tests except for the Stroop interference ratio. Moreover, Table 3 shows that, across both tests and dimensionalities, PCA-based representations perform better than population average atlases in 66.4% of cases. Summarizing results per dimensionality, Table 4 shows that PCA-based representation performed better than population average atlases on more than half of the cognitive test scores for 8 out of the 13 atlases (61.5%). Moreover, on average, PCA-based representations resulted in better predictions on 5.31 out of 8 cognitive tests**.** This shows that PCA-based representations were more suitable to predict cognitive functioning when compared to population average atlases.
7. Given an atlas, would a different representation with the same number of dimensions result in better predictions?
  Table 5 shows which representation results in the best predictions given a cognitive test and dimensionality. Population average atlases were the best representation for 27 out of 104 (26.0%) combinations of dimensionality and cognitive test. Random atlases made up 30 (28.8%) of the best-performing representations, and PCA-based representations made up 47 (51.9%) of the best-performing representations. These results show that PCA-based representations most often resulted in the best performance when compared to atlases and random atlases with the same number of dimensions. Moreover, this shows that random atlas-based representations more often resulted in the best performance when compared to population average atlases, although the difference is small.

**Table 5:** Best-performing representation given a cognitive test and dimensionality for which there is an atlas included. Population average atlases are displayed in bold to make them easily distinguishable from other representations

*Number of dimensions / regions*	*Continuous performance test*	*Finger tapping test*	*Shifting attention test*	*Simple reaction time*	*Stroop interference ratio*	*Symbol digit coding test*	*Verbal memory recognition*	*Visual memory recognition*
*2*	**Hemisphere**	**Hemisphere**	Random Atlas 2	**Hemisphere**	PCA 2	**Hemisphere**	PCA 2	PCA 2
*7*	PCA 7	PCA 7	**Yeo 7 networks**	PCA 7	PCA 7	PCA 7	Random Atlas 7	PCA 7
*9*	PCA 9	**MNI maxprob**	Random Atlas 9	PCA 9	PCA 9	PCA 9	Random Atlas 9	PCA 9
*17*	PCA 17	PCA 17	**Yeo 17 networks**	PCA 17	PCA 17	Random Atlas 17	Random Atlas 17	PCA 17
*18*	PCA 18	PCA 18	Random Atlas 18	PCA 18	PCA 18	Random Atlas 18	Random Atlas 18	PCA 18
*96*	PCA 91	PCA 91	Random Atlas 91	**OASIS DKT**	**OASIS DKT**	Random Atlas 91	Random Atlas 91	**OASIS DKT**
*100*	PCA 100	PCA 100	**Schaefer 100**	PCA 100	**Schaefer 100**	Random Atlas 100	Random Atlas 100	Random Atlas 100
*113*	Random Atlas 113	PCA 113	Random Atlas 113	**HarvardOxford**	**HarvardOxford**	Random Atlas 113	**HarvardOxford**	**HarvardOxford**
*166*	PCA 166	Random Atlas 166	PCA 166	PCA 166	Random Atlas 166	Random Atlas 166	PCA 166	PCA 166
*200*	PCA 200	**Schaefer 200**	Random Atlas 200	PCA 200	**Schaefer 200**	Random Atlas 200	PCA 200	PCA 200
*246*	Random Atlas 246	**Brainnetome**	PCA 246	**Brainnetome**	Random Atlas 246	Random Atlas 246	**Brainnetome**	Random Atlas 246
*300*	PCA 300	**Schaefer 300**	PCA 300	**Schaefer 300**	PCA 300	**Schaefer 300**	PCA 300	Random Atlas 300
*360*	**MMP**	Random Atlas 360	PCA 360	**MMP**	**MMP**	PCA 360	PCA 360	Random Atlas 360

## Discussion

Overall, the performances of different representations were largely similar, and using representations of tumor location did not always result in better predictions when compared to the baseline model which used tumor volume as a predictor (question 3). Using population average atlases did not result in better predictions of pre-operative cognitive functioning in patients with high-grade glioma when compared to random atlases (question 4). PCA-based representations did not clearly outperform other representations, although summary metrics indicated that PCA-based representations performed slightly better than population average atlases (questions 2, 3, 6, and 7) and random atlases (questions 2, 3, 5, and 7).

Population average atlases not resulting in better predictions when compared to random atlases (question 4) is somewhat surprising given that regions in population average atlases are expected to have a certain level of functional distinctiveness. This finding may be explained by the mass effect of the tumor limiting the accuracy of population average atlases. Brain tumors, including glioma, generally compress surrounding tissue causing deformations (visualized in Appendix 5). This mass effect may compromise the accuracy of the parcellation resulting from registering a population average atlas to scans of patients with a brain tumor for two reasons. First, both the presence of the tumor and the aforementioned deformations may cause inaccuracies when registering the patient scan to a common space (Visser et al., 2020). These inaccuracies in registration cause the atlas as a whole to line up incorrectly, causing inaccuracies across *all regions*. Second, the presence of the tumor and the resulting compression of nearby tissue can cause certain brain regions to shift relative to the rest of the brain, leading to misalignment of these *specific shifted regions*.

Alternatively, this finding may be explained by individual differences in (functional) anatomy (Kanai & Rees, 2011), which are not taken into account by the current parcellation method. This may cause regions that are functionally distinct at the group level to not be sufficiently functionally distinct for individuals to aid prediction performance. Moreover, this finding may be explained by neuroplasticity, which refers to the brain’s ability to reorganize itself to compensate for functional impairments caused by the tumor (Dąbrowski et al., 2019; Duffau, 2017; Lv et al., 2022). Neuroplasticity may cause regions that are functionally distinct in a healthy population to not be functionally distinct for a given patient with a brain tumor.

There was no clear pattern in the characteristics of the population average atlases that performed best (questions 2 and 3): the dimensionality of these different atlases ranged from 2 to 113, they were based on structure or functional connectivity, and were either volumetric or surface-based. This indicates that the performance of these atlases is due to chance instead of due to some common property. When comparing the best-performing atlases between different cognitive tests, this heterogeneity may also be explained by certain characteristics being better suited when predicting a specific cognitive function, which is in line with the idea that the best functional atlas depends on the cognitive function of interest (Salehi et al., 2020).

PCA-based representations did not clearly outperform other representations. However, according to the summary metrics, PCA-based representations did perform somewhat better when compared to population average atlases (supported by all questions addressing this difference: questions 2, 3, 6, and 7) and random-atlases (supported by questions 2, 3 [in part] and 7, but not by question 5) in our sample. This indicates that PCA-based representations may be more suitable than population average atlases or random atlases when predicting cognitive functioning of patients with a high-grade glioma across cognitive tests. However, it is important to note that the differences found in the current study were small. Therefore, future studies should test if this small advantage as found in the current study generalizes to other data. Unlike atlases where each region is clearly defined and interpretable, PCA-based representations are somewhat less interpretable. Therefore, researchers aiming to obtain the highest predictive can consider using PCA-based representations if this decrease in interpretability is acceptable given the goal of the predictions.

Several characteristics of PCA may explain why PCA-based representations performed somewhat better when compared to population average atlases. First, PCA-based representations are not confined to predefined regions that may be inaccurate. Instead, the eigenvectors found by PCA solely depend on the correlations amongst voxels. Second, PCA-based representations more efficiently capture the variance in the data compared to atlases, requiring fewer features to describe the same amount of information. Last, variables in the PCA-based representation are uncorrelated which improves the numeric stability of regression models whereas overlap with regions in an atlas are correlated due to spatial continuity. Due to differences in methodologies, we were unable to compare the predictions of cognitive functioning to those reported by Zangrossi et al. (2022) who also represented tumor location using PCA.

Representations based on PCA did not always result in better performance when compared to the baseline model (questions 3 and 5), as was the case for atlas and random atlas-based representation. Similar to atlas-based representations, this may be explained by individual differences in anatomy, mass effects, and registration errors. These individual differences, mass effects, and inaccuracies in registration may cause voxels in MNI space to refer to slightly different parts of the brain for different patients. This limits the meaning of individual voxels in MNI space and thus the resulting components as found using PCA based on the correlations amongst these voxels.

The first three PCA components found in the current study roughly described the distinction between the frontal, parietal, and temporal lobes. This is in line with the work of Mandal et al. (2021) who found a similar distinction using independent component analysis. In our study, however, these regions were not completely separated across components as was in their work, which is expected given the difference in methodology. Furthermore, our first three components are partly in line with those of Zangrossi et al. who performed PCA in a sample of 47 high- and low-grade gliomas (Zangrossi et al., 2022). Their results, however, differ in that they found a temporoparietal component instead of a parietal component. Furthermore, their first three components explained 55% of the variance instead of 20% as found in the current study. These differences can likely be attributed to the smaller sample size in their study.

The baseline model that uses only tumor volume explained up to over 7.9% of the variance depending on the cognitive tests (question 1). This result is in line with previous studies that found significant relationships between tumor volume and cognitive function across multiple cognitive domains (Karunamuni et al., 2020) and with studies finding significant relationships for some, but not all cognitive tests (Noll et al., 2016; van Kessel et al., 2019). Furthermore, representations of tumor location improved predictions when compared to the baseline model for some, but not all test variables (question 3). This may be explained by some measures drawing more from localized functions or functional hubs instead of more distributed functions when compared to other tests (Sepulcre et al., 2010). Moreover, this is in line with lesion-symptom mapping studies in patients with a brain tumor finding significant regions for some, but not all cognitive tests (De Baene et al., 2019a, b; Habets et al., 2019).

Representations of tumor location explained at most 15.1% of the variance in test scores in addition to the baseline model (question 2). This limited amount may again be explained by individual differences in (functional) anatomy or neuroplastic processes which results in functions being in different regions for different patients, limiting predictions regardless of the representation used. The amount of variance that can be explained for individual tests is further limited by the test–retest reliability, which differs per test and sample tumor (Gualtieri & Johnson, 2006; Rijnen et al., 2018). Unfortunately, this information is not available for patients with a brain tumor. Finally, the amount of variance that can be explained is limited by a large number of other factors besides tumor location and volume that influence cognitive functioning (Boelders et al., 2023). This includes variables such as histopathology (Karunamuni et al., 2020; Noll et al., 2015; van Kessel et al., 2017, 2019, 2022), patient characteristics (Derks et al., 2018; Pranckeviciene et al., 2020; van Kessel et al., 2022), anxiety and depression (Tibbs et al., 2020), and medicine use (de Groot et al., 2013; Karunamuni et al., 2020; Morshed et al., 2021).

Results showed that representations with high dimensionality resulted in less accurate predictions, which is in line with the curse of dimensionality (Mwangi et al., 2014). This finding may in part be due to the sample size. Larger sample sizes, however, are uncommon for research with patients with primary brain tumors. Given that using smaller regions in an atlas increases the errors relative to the size of the regions, and that using more PCA components causes these representations to consider smaller clusters of voxels that less accurately map back to the patient's anatomy, these results lead us to recommend researchers to use representations with fewer regions (up to roughly 91 regions, see questions 2 and 3). These findings are in line with a recent study by Litwińczuk et al. (2024) who found that parcellation schemes with fewer regions most consistently resulted in performance above change levels when predicting cognitive functioning of healthy participants based on structural and functional connectivity.

A few limitations of the study should be mentioned. First, the current sample was collected as part of clinical practice and does not include patients who were excluded from neuropsychological testing, for example, due to too severe cognitive deficits, motor problems, or because of the need for immediate surgery. This could have led to a small overestimation in test performance. Second, the cognitive tests performed in the current study were part of a brief computerized test battery that does not cover functions such as visuoconstructive ability, language, and memory free recall. Hence, the present research does not eliminate the possibility that there are cognitive functions for which the relationship with the representations of tumor location differs from the current findings. More extensive cognitive assessments, however, generally are not performed in presurgical clinical practice for patients with brain tumors at a large scale. Last, its tests may be somewhat dependent on processing speed (Gualtieri & Hervey, 2015).

It should also be noted that the current study aimed to test the predictability of cognitive functioning for unseen patients using different representations of tumor location, uncovering how informative each representation is for such predictions. This differs from explanatory modeling where one tries to model the underlying causal structure by assessing model fit (Shmueli, 2010). Given the large number of models compared, we were unable to perform any statistical tests regarding prediction performance. Therefore, different summary metrics to describe trends in model performance were calculated instead.

We believe our results to be highly relevant as they show that preoperative cognitive functioning can only partly be predicted using representations of tumor location as commonly used. Post-operative functioning is influenced by effects of the residual tumor and of surgery and adjuvant treatment (Butler et al., 2006; Li & Caeyenberghs, 2018) in addition to the damage caused by the tumor before surgery which is predicted in the current study. Moreover, both the treatment and the residual tumor depend on the tumor location before surgery. Therefore, it is likely more difficult to find a representation that allows for predicting post-operative cognitive functioning. This is in line with the study Zangrossi et al. (2022) who found that tumor location before surgery did not improve prediction models of post-operative cognitive functioning.

We further believe our results to be important as they show that using representations of tumor location based on registering a population average atlas to a common space may not allow for better predictions of cognitive functioning compared to random atlases. This stresses the need for better methods to automatically parcellate the brain in the presence of a lesion. This is especially important given that datasets are becoming increasingly large, which makes the manual labeling of tumor involvement with different areas laborious. Our results further indicate that researchers who aim to predict cognitive functioning using tumor location could use a PCA-based representation over atlas-based representations if the decrease in interpretability is acceptable. Finally, the current study further suggests that researchers using atlases may be better off when using population average atlases with fewer regions as compared to using more detailed regions which increases the inaccuracy of the parcellation without allowing for better predictions.

To improve methods for automatic parcellation of the brain in the presence of lesions, research can consider validating methods such as virtual brain grafting (Radwan et al., 2021) where a lesion-free image is generated from the pathological image after which traditional parcellation techniques can be applied. Moreover, deep learning models can be developed to parcellate the brain in the presence of brain tumors, such as recently done for healthy participants (Billot et al., 2023; Huo et al., 2019), although we recognize the challenge of obtaining sufficient labeled data required to train such models. Last, mathematical models of mass effects such as the ones performed by GLISTR (Gooya et al., 2012; Subramanian et al., 2023) can be considered to transform voxel-wise segmentations into a space that is corrected for mass effects before applying population average atlases. Such a space may more accurately represent the healthy tissue that would have been located at the voxels that make up the tumor.

In addition to improving methods for obtaining parcellation in the presence of a lesion, researchers can consider using different methods to create lower-dimensional representations of voxel-wise tumor location. For example, non-linear dimension reduction techniques including manifold learners such as tSNE (Van der Maaten & Hinton, 2008; van der Maaten et al., 2007) can be used. Alternatively, it may be possible to use variational auto-encoders to learn a joint representation that considers both the voxel-wise tumor involvement and the anatomical scan depicting individual differences and mass effects (Doersch, 2016). Future research can further test if using atlases that focus on a specific function of interest allows for better predictions of this function when compared to the atlases presented in the current study. Moreover, researchers should test if the choice of an atlas matters when predicting cognitive functioning of patients with a brain tumor from structural or functional connectivity. Finally, future research can consider including a representation of the region affected by edema in addition to the tumor segmentation, as this is likely to affect cognitive functioning as well (Dallabona et al., 2017).

## Conclusion

Preoperative cognitive functioning could only partly be inferred from the representations of tumor location as commonly used. Therefore, it is unlikely that such representations can explain a large amount of variance when predicting post-operative cognitive functioning. The performances of different representations were largely similar and population average atlases did not result in better predictions when compared to random atlases. This indicates that the regions in population average atlases do not distinguish between functionally distinct areas when applied to patients with a high-grade glioma. Moreover, this stresses the need to develop and validate methods for individual parcellations in the presence of lesions. PCA-based representation did not clearly outperform other representations. However, the small advantage of PCA-based representations as observed for our sample may prompt future research to test if this difference generalizes to new data. Finally, results showed that using atlases with more regions resulted in less accurate predictions.

## Information Sharing Statement

The current patient samples are described (in part) in previous studies (Boelders et al., 2023; Butterbrod et al., 2019, 2020, 2021; De Baene et al., 2019a, b; Lonkhuizen et al., 2019; Meskal et al., 2015; Rijnen et al., 2019, 2020a, b; van der Linden et al., 2020; van Loenen et al., 2018). This project was part of a study protocol registered at the Medical Ethics Committee Brabant (file number NW2020-32).

### Supplementary Information

Below is the link to the electronic supplementary material.

Supplementary file1 (PY 28 KB)

Supplementary file2 (PNG 191116 KB)

Supplementary file3 (XLSX 466 KB)

## Figures and Tables

**Figure:**
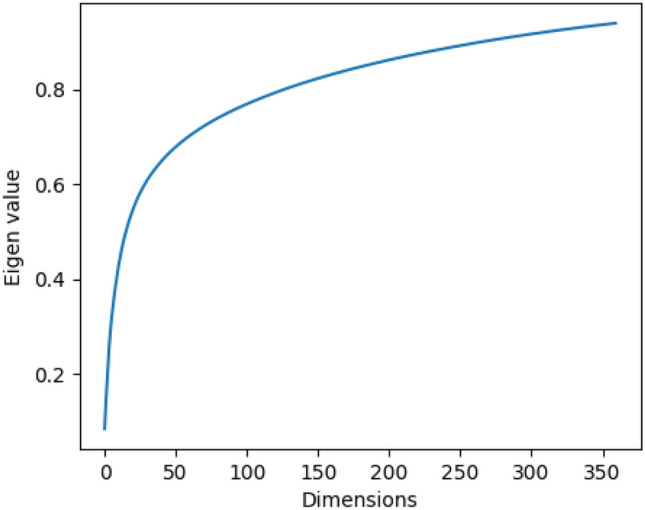
Cumulative amount of variance explained versus the number of dimensions used by PCA

**Figure:**
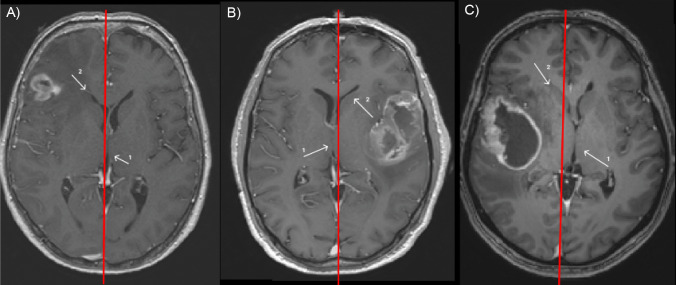
Example of the mass effect of a **A** small, **B** medium, and **C** large glioma. 1) shows the midline shift where the midline is shown as a red line, 2) shows the compression of the ventricles.

## Data Availability

Data used in this study is not publicly available to protect the privacy of the patients. All code used for analysis is made available as an online supplement.

## Appendix 1: CNS VS Tests

**Table 6:** Calculations of cognitive tests scores

Name	Description	Formula
Verbal memory recognition test	Memory recognition for words. Fifteen words are presented one at a time. The subject identified the presented words amongst new words. The immediate condition is at the beginning of the test battery and the delayed condition is at the end.	Number of items correct (immediate + delayed condition)
Visual memory recognition test	Memory recognition for abstract images. Fifteen images are presented one at a time. Subjects identified the presented images amongst new images. The immediate condition is at the beginning of the test battery and the delayed condition is at the end.	Number of items correct (immediate + delayed condition)
Symbol digit coding test	Eight symbols are presented on the screen with a corresponding number. Given a row of eight randomly ordered symbols, the participant is asked to provide the matching number for two minutes straight.	Correct responses - incorrect responses
Simple reaction time	The subject presses the space bar when a word is presented.	Average reaction time
Stroop test interference	The subject presses the space bar if a word is presented and the color of the word does/or does not match its semantic meaning for the congruent and incongruent trials respectively	(Average reaction time on correct responses for the incongruent trials - Average reaction time on correct responses for the congruent trials) / Average reaction time on correct responses for the congruent trials
Shifting attention test	A red circle and a blue square are presented on the screen. Given a third shape, the participant needs to match the shape either by color or shape.	Correct responses - incorrect responses
Continuous performance test	The subject responds to a target letter amongst distractors for 5 minutes straight.	Average reaction time to the target letter
Finger tapping test	The participant presses the spacebar as often as possible within ten seconds. This task is performed three times for each hand.	The average number of presses across left + right trials

## Appendix 2: Included Atlases and Their Characteristics

**Table 7:** Atlases included in the current study and their characteristics

**Name**	**Type**	**Volume covereed by the atlas (mm**^**3**^**)**	**Number of regions**	**Includes subcortical structures**	**Reference**
AAL3v1	Structure	1486008	166	yes	(Tzourio-Mazoyer et al., 2002; Rolls et al., 2020)
HarvardOxford	Structure	1173803	69	yes	N.A
Brainnetome	Multimodal	1124225	246	yes	(Fan et al., 2016)
OASIS DKT	Structure	893505	109	no	(Desikan et al., 2006; Klein and Tourville, 2012; Klein et al., 2017)
MMP	Multimodal	629569	380	no	(Glasser et al., 2016)
Schaefer	rsfMRI	1055685	100 / 200 / 300	no	(Schaefer et al., 2018)
Yeo networks	rsfMRI	1054733	Jul-17	no	(Yeo et al., 2011)
Random atlases	N.A.	2142930	N.A.	yes	(Zalesky et al., 2010)
Hemisphere*	Structure	2142930	2	yes	N.A
MNI maxprob*	Structure	2142930	9	yes	(MacDonald et al., 2000; Mazziotta et al., 2001)
MNI maxprob per hemisphere*	Structure	2142930	18	yes	N.A

For the Schaefer and Yeo networks atlases, multiple version were included with different numbers of regions

*Atlases are not part of the standard set of atlases by Revell and colleagues (2022)

## Appendix 4: Comparisons per Number of Dimensions While Including the Dimensionalities Used for Visualization (Similar to Table 4)

**Table 8:** Results summarized over the eight different tests

**Table 4: Comparisons per number of dimensions**							
		Question 3			Question 4	Question 5	Question 6
**Number of dimensions**	Atlas	Random atlas better than baseline	Population average atlas better than baseline	PCA better than baseline	Population average atlas better than random atlas	PCA better than random atlas	PCA better than population average atlas
**2**	Hemisphere	**5**	4	3	5	2	4
**3**		**5**		2		2	
**7**	Yeo 7 networks	**6**	4	**6**	2	**5**	**7**
**9**	MNI maxprob	**6**	4	**6**	2	**5**	**7**
**10**		**5**		**6**		**5**	
**12**		**6**		**5**		**5**	
**14**		**5**		**5**		**4**	
**17**	Yeo 17 networks	**6**	**5**	**5**	3	**5**	**6**
**18**	MNI maxprob per hemisphere	**5**	4	**5**	2	**5**	**7**
**21**		**5**		**6**		**6**	
**25**		**5**		**5**		**5**	
**31**		**5**		**7**		**6**	
**37**		**5**		**5**		**6**	
**45**		**5**		4		4	
**54**		**5**		3		3	
**65**		**5**		3		3	
**79**		4		3		3	
**91**		4		4		3	
**96**	OASIS DKT	4	3	3	4	3	**5**
**100**	Schaefer 100	4	3	4	3	3	**5**
**113**	HarvardOxford	4	4	4	4	2	4
**116**		**5**		4		2	
**140**		4		4		4	
**166**	AAL3v1	3	1	4	2	**5**	**7**
**169**		4		4		4	
**200**	Schaefer 200	3	1	3	3	4	**6**
**204**		3		3		**5**	
**246**	Brainnetome	3	1	3	3	2	4
**298**		3		3		4	
**300**	Schaefer 300	3	3	3	3	3	3
**360**	MMP	2	3	3	4	**5**	4
Average		**4.42**	3.08	**4.13**	3.08	3.97	**5.31**

The number of cognitive tests on which a representation of tumor location resulted in a larger amount of variance explained when compared to some other representation of tumor location, individually for each dimensionality. Representations that outperformed some other representations on more than half of the tests are displayed in bold
